# Thyroid Dysfunction among Greek Patients with Type 1 and Type 2 Diabetes Mellitus as a Disregarded Comorbidity

**DOI:** 10.1155/2017/6505814

**Published:** 2017-10-30

**Authors:** Maria E. Barmpari, Maria Kokkorou, Anastasia Micheli, Irene Alexiou, Elefteria Spanou, Marina Noutsou, Anastasia Thanopoulou

**Affiliations:** Diabetes Center, 2nd Department of Internal Medicine, Medical School, National and Kapodistrian University of Athens, Hippokration General Hospital of Athens, Athens, Greece

## Abstract

**Introduction:**

The aim of this study was to determine the prevalence of thyroid dysfunction in Greek patients with type 1 (T1DM) and type 2 (T2DM) diabetes mellitus as well as its possible relations to glycaemic control and to diabetic complications.

**Methods:**

A total of 1015 patients, consecutively followed in the Outpatient Diabetes Center, were studied. Anthropometric and biochemical measurements, occurrence of diabetes complications, and classical comorbidities were assessed. Average HbA1c of the previous year was calculated. Wellbeing was determined, using a 10-point optimal scale. All the above parameters were compared between subjects with or without thyroid disease.

**Results:**

All patients were euthyroid at the time of the study, either on thyroid medications or not. Hypothyroidism occurrence did not differ between T2DM and T1DM patients (37.1% versus 43.5%, *p* > 0.05). Nodular goiter was observed more frequently in T2DM patients (34.1% versus 18.8%, *p* < 0.05). T2DM patients with hypothyroidism compared to those without hypothyroidism had higher HbA1c (7.27% versus 6.98%, *p* < 0.01), TChol (184.97 mg/dl versus 168.17 mg/dl, *p* < 0.001), and higher HDL-Chol (51.28 mg/dl versus 46.77 mg/dl, *p* < 0.01). T2DM patients without hypothyroidism had a better wellness feeling (7.5 versus 5.3 points, *p* < 0.01).

**Conclusions:**

Screening for thyroid disease among T2DM patients should be routinely considered, as it is found to be an additional commorbidity. If it remains undiagnosed, it could aggravate the clinical course of the disease.

## 1. Introduction

Diabetes mellitus and thyroid diseases represent two of the most common endocrinopathies [1]. In 2014, 8.2% of adults aged 20–79 y (387 million people) were living with diabetes according to the International Diabetes Federation's data [2]. In Greek population, during 10 years of follow-up (2002–2012), there was a 12.9% incidence of type 2 diabetes mellitus (T2DM) with an increase in prevalence among individuals more than 65 years old (ATTICA study) [3].

Thyroid dysfunction includes hypothyroidism (Hashimoto's thyroiditis), hyperthyroidism (Grave's disease), and diffuse nodular goiter. The first two endocrinopathies are induced by an autoimmune process, and they are included in the spectrum of autoimmune thyroid disease (AITD).There is a large variability between studies in the general population, concerning the prevalence of overt or subclinical hypothyroidism ranging from 2–4% to 4–20%, respectively, rising significantly in women >60 years old [4–7]. Subclinical hypothyroidism is the most common endocrinopathy among the different studies [5–9]. In contrast, the prevalence of overt or subclinical hyperthyroidism is much lower 0.3% and 1%, respectively [4–7].

Among patients with diabetes, the prevalence reported varies from 13.4% in Scotland to 12.5% in Jordan and from 15% in Spain to 26% in Saudi [10–14]. The discrepancy of these results could be explained by the different definitions used for the diagnosis of thyroid dysfunction which is supported by the presence of only one or both of the two thyroid autoantibodies (antithyroid peroxidase anti-TPO, antithyroglobulin antibodies anti-TG).When hypothyroidism is caused by autoimmune thyroiditis, which is its prevalent cause, it shares a common genetic origin with T1DM [15]. On the other side, interrelationships between T2DM and thyroid dysfunction have also been suggested but the underlying mechanisms are more complex [16]. In Greece, the prevalence of thyroid disease in T2DM patients was reported to be 12.3% [6].

There are no enough population studies about the presence of nodular goiter among patients with diabetes. Etiologic factors such as iodine deficiency, smoking, and genetic factors represent the main causes for the formation of thyroid nodular goiter. In adults, there is a lack of data on the comorbidity of T1DM and thyroid nodularity because the majority of studies have included children and adolescents. Currently available data demonstrated conflicting results in the presence of thyroid goiter in T1DM patients [17–19]. In T2DM patients, there are some studies in which the prevalence of T2DM and nodular thyroid goiter was 61.8% in a mild to moderate iodine deficient area [20–22]. The main etiologic factor in the previous study was insulin resistance [23–25].

## 2. Patients and Methods

This is a single-center study including euthyroid patients with T1DM and T2DM, consecutively followed in our Outpatient Diabetes Clinic. A total of 1015 patients (60.4% men), with T1DM or T2DM, participated in the study. All patients were ambulatory without a recent acute illness. They had been diagnosed with T1DM or T2DM according to the American Diabetes Association criteria. Patients taking drugs responsible for thyroid dysfunction were excluded from the study. The study was performed in a sufficiently iodized area, Athens, Greece. All patients gave informed consent before the study.

In every patient, anthropometric data (weight, height, waist and hip circumference, and body mass index (BMI)) duration of diabetes, occurrence of diabetic complications and classical commorbidities (hypertension and dyslipidemia), diabetes' treatment, and biochemical measurements (renal and liver function tests) were recorded. Average HbA1c of the previous year was calculated. We have included in the study the last hormonal tests of the patients (done not earlier than 6 weeks) that included TSH, T3, and T4 levels, and all were in the normal reference range of the laboratory in which they were conducted. Body weight was measured with light clothing without shoes, and body mass index (BMI) was calculated. Blood pressure was recorded as the mean of three consecutive measurements taken 5 min apart and then in the sitting position. Moreover, the wellbeing of every patient was determined, using a scale from 1 to 10. This was an optic wellness scale performed only for this study by all the patients included. The patients, with and without hypothyroidism, were questioned about their wellness feeling, and they were called to grade it from 1 to 10. All the above parameters were compared between subjects with or without thyroid disease.

Thyroid ultrasonography had been performed in every patient, in Hippokration General Hospital of Athens with the same operator. Only patients with nodular goiter were included in the study and not those with a diffuse goiter. Patients with a single thyroid nodule and those with diffuse multinodular goiter were considered together. Some of these patients were under thyroxine substitution because of the coexistence of hypothyroidism. Hypothyroidism was considered as the presence of thyroid autoimmunity and the use of thyroxine substitution. Thyroid autoimmunity was considered when thyroid autoantibodies against thyroid peroxidase (anti-TPO) were positive. The patients with a history of thyroidectomy or use of iodine therapy for hyperthyroidism were excluded from the study. There were so few patients with a history of hyperthyroidism that were not included in the study. All the patients included were euthyroid with or without thyroxin substitution during the period in which the study was conducted.

## 3. Statistical Analysis

For quantitative variables, results are expressed as mean ± SD for parametric data and as median for nonparametric data. Categorical variables are described as percentages (%). Differences were considered significant when *p* < 0.05. Statistical analysis was performed using the SPSS 20.0 Package. A two sample *t*-test was used to assess differences in continuous variables. For categorical variables, *x*-squared was performed.

## 4. Results

From the 1015 patients, 12.6% (58.4% men) had T1DM with a mean age of 47.5 ± 1.4 years and a mean duration of diabetes 23.6 ± 1.1 years. The rest 87.4% (52.8% men) with a mean age of 67.5 ± 0.3 years and a mean duration of diabetes 15.9 ± 0.3 years had T2DM [Table 1].

No statistically significant difference was observed in the prevalence of hypothyroidism between T1DM patients (43.5%) and T2DM patients (37.1%). On the other hand, thyroid nodular goiter was more frequent in T2DM patients (34.1% versus 18.8%, *p* < 0.05) compared to T1DM patients even after age adjustment [Table 1]. There was no statistically significant difference among T2DM patients with nodular goiter in therapy with antidiabetic agents and those with agents plus insulin.

BMI was significantly higher in patients with thyroid nodular goiter compared to those without, even after age adjustment (30.1 ± 0.411 versus 28.7 ± 0.283, *p* < 0.01).

In T1DM patients, there were no statistically significant differences between the patients with hypothyroidism and those without, except from HDL cholesterol levels that were higher in the patients with hypothyroidism [Table 2].

In T2DM patients, there was a higher prevalence of hypothyroidism between females (*p* < 0.001) [Table 3]. Patients with T2DM without hypothyroidism had better glycaemic control with glycosylated haemoglobin levels below 7%, compared to those with abnormal thyroid profile [Table 3]. Moreover, patients with T2DM and hypothyroidism had higher total cholesterol and HDL cholesterol levels compared to patients without [Table 3]. Finally, T2DM patients without hypothyroidism feel significantly better than those with hypothyroidism [Table 3].

No statistically significant difference was observed in biochemichal and antropometric measurements between the patients with nodular goiter and those without.

Additionally, in both types of diabetes mellitus, there were no statistically significant differences between all the patients with and without hypothyroidism and those with and without nodular goiter and the occurrence of diabetes complications (liver and renal function tests (data not shown).

## 5. Discussion

The interactions between diabetes mellitus and thyroid diseases are well known during the last decades [5]. Hypothyroidism and T1DM share a common pathophysiology because of the presence of autoimmunity in both diseases [8]. Population studies describe a common genetic basis for thyroid disease and diabetes mellitus, with the strongest association between the latter and autoimmune thyroiditis [8]. Among T1DM patients, thyroid antibodies may reach 50% and progression to overt hypothyroidism is more than possible [26]. Thus, the high prevalence of hypothyroidism in T1DM patients found in our study is expected. In contrary, the interrelationship between T2DM and hypothyroidism is not yet well defined. Few studies describe a common genetic origin among the diseases based on a polymorphism in the gene of type 2 deioidinase [27]. As discussed by Celani et al., subclinical hypothyroidism has the highest prevalence among DM2 patients, followed by overt hypothyroidism [28]. In the study of Udiong et al., hypothyroidism is the commonest disease in T2DM patients [29]. In the present study among 1015 diabetic euthyroid patients, the prevalence of hypothyroidism in T2DM patients is comparable with the respective of T1DM patients [Table 1]. This is an important finding of this study because in patients with T2DM the etiology of hypothyroidism is not well known. The cause of hypothyroidism in T2DM patients in our study is autoimmunity as well as in T1DM, because only patients with autoimmunity were included in the study.

The second interesting finding of the present study is the high prevalence rate of thyroid nodular disease in T2DM patients that differs significantly from those with T1DM. There are conflicting results related with the presence of goiter in T1DM patients [8, 9]. In those patients, the main factor of thyroid goiter is autoimmunity but the majority of the studies were conducted in children and adolescents [15–18]. Additionally, iodine deficiency of the area results in an increase of serum TSH which represents the major regulator of the growth and the thyrocyte's differentiation and proliferation [30, 31]. However, TSH alone could not explain the formation of nodular goiter because it is not always in higher levels in cases of nodularity, as it is supported in the study of Junik et al. [20]. Our study is conducted in Athens, Greece, a sufficiently iodine-supported area after the introduction of iodine in salt [32].

There is a variety of factors reported in different studies, as the possible causes of thyroid nodularity such as smoking, iodine deficiency, and genetic factors [33, 34]. Recent studies suggest an important role of insulin resistance in the formation of nodular goiter [24]. It is well known that insulin and insulin growth factors (IGFs) exert a mitogenic effect on cell cultures increasing cell proliferation. IGF-1 and IGF-2/insulin signaling pathways have been shown to modulate regulation and expression of thyroid genes [35]. By this way, insulin resistance and compensatory hyperinsulinaemia may participate in thyroid cell proliferation and thyroid nodular formation [36]. Population studies describe a higher thyroid volume and a major incidence of thyroid nodular goiter measured on ultrasound in subjects with hyperinsulinaemia and metabolic syndrome [36]. It is well known that metabolic syndrome is more frequently presented in obese patients. TSH levels were positively correlated with body mass index (BMI) in the study of Anil et al., which had also found higher TSH levels in T2DM patients than in controls and those with prediabetes [23]. In the present study, patients with nodular goiter had higher body mass index (BMI) after age adjustment. The higher prevalence of thyroid nodular goiter in T2DM patients may be explained by the same higher prevalence of obesity in these patients with respect to those with T1DM. In any case, insulin resistance is not the only possible cause of thyroid nodularity [33, 34].

Different clinical aspects are described in conditions of excess or insufficient secretion of thyroid hormones [37]. In hypothyroidism, there is a slow glucose gastrointestinal absorption, retardation of gluconeogenesis, and glucose utilization peripherally with a consequent increase of insulin resistance [38, 39]. In the present study, T1DM patients with hypothyroidism had comparable HbA1c levels to patients without hypothyroidism [Table 2]. On the other hand, T2DM hypothyroid patients had worst glycaemic control with higher HbA1c levels in comparison to those without hypothyroidism [Table 3]. Additionally, in both types of diabetes mellitus, there were no statistically significant differences between all the patients with and without hypothyroidism and those with and without nodular goiter and the occurrence of diabetes complications and biochemical parameters.

There are central and peripheral effects of thyroid hormones on glucose metabolism and lipid metabolism regulated by insulin secretion and insulin sensitivity on different tissues [40, 41]. From our study arises a statistically significant increase in HDL cholesterol levels in hypothyroid T1DM patients [Table 2]. Hypothyroid patients may exhibit elevated HDL cholesterol levels mainly due to increased concentration of HDL particles, but also because of the decreased activity of cholesteryl ester transfer protein (CETP) [42]. In the present study, the same statistically significant difference with higher total cholesterol and HDL levels is presented in T2DM hypothyroid patients in respect of those with normal thyroid profile [Table 3]. During the study, all the patients were euthyroid. L-thyroxine replacement therapy tends to improve lipid profile in hypothyroid patients after 4–6 weeks of substitution, even if qualitative studies on LDL particle size did not assess any changes and HDL cholesterol levels did not decreased significantly [43]. We do not know exactly what period is needed so that total cholesterol and HDL levels come into the normal reference ranges after thyroxine substitution [43].

Because of the high prevalence of thyroid autoimmunity in T1DM patients, they have to be controlled every year for thyroid disorders as it is recommended by the American Diabetes Association guidelines [44]. For T2DM patients, the American Thyroid Association guidelines suggest frequent testing for adults over 35 years and every 5 years thereafter. Thyroid Disease Clinical Practice Guidelines (2002) and the American Association of Clinical Endocrinologists suggest a control of TSH levels in diagnosis and periodic controls in case of autoimmune disease or goiter in palpation [44]. In a recent population prospective cohort, study concluded that low–normal thyroid function represents an important risk factor of developing diabetes in patients with prediabetes [45]. All the above guidelines agree with the periodic control of the thyroid function, and we would like to superinduce the ultrasound of thyroid as a necessary mean to discover nodular goiter, after the palpation of the gland.

Metformin therapy reduces TSH levels in DM and hypothyroid patients, possibly by its action in the hypothalamus [46]. There are studies that support that metformin has a suppressive action in TSH levels, without interfering with thyroid hormone levels. This effect is independent of thyroid autoimmunity and thyroxine treatment [46]. Even if Rezzonico et al. showed a reduction in nodular size after metformin therapy in patients with insulin resistance and small thyroid nodular goiters, that was not confirmed in larger clinical studies [47, 48]. This is confirmed from our study too, because the majority of our T2DM patients is under metformin therapy, but continues to have a major prevalence in thyroid nodular goiter. Metformin has a neutral effect on goiter, whereas insulin therapy possibly exerts a protective role against the formation of nodular goiter. In one study conducted in adults, T1DM patients showed a lower incidence of thyroid nodular goiter compared to those with T2DM [9, 48].

Thyroid dysfunction is generally considered as a disregarding commorbidity of diabetes mellitus. In the present study, we would like to highlight the importance of hypothyroidism in T2DM patients that tend to reach the same prevalence as those with T1DM. Hypothyroidism will alter glycaemic and lipid profiles of the patients with prediabetes and will aggravate the course of the disease in those with already known diabetes [45]. We suggest more often measurements of thyroid hormone levels in T2DM patients and even in those with prediabetes.

Additionally, thyroid nodular goiter is a frequent comorbidity in T2DM patients but also in those with T1DM, even in a lower prevalence rate. The palpation of the gland is not enough for the detection of thyroid nodular goiter especially for nodules under 1 cm in diameter. In any case, an ultrasound of the thyroid could be necessary for the detection and the monitoring of these nodules. It is important to discover it as soon as possible because of the continually increasing prevalence of thyroid cancer and microcarcinoma of the thyroid but also because of the presence of toxic thyroid nodular goiter more frequently in the elderly [49, 50]. Hyperthyroidism provocated from toxic nodular goiter is responsible for increased insulin degradation, increased glucagon secretion, and increased hepatic glucose production, all these factors which aggravate insulin resistance and glyceamic control of the patients with diabetes. It is important to diagnose and cure it as soon as possible [51]. On the other hand, in case of noncontrolled hypothyroidism or hyperthyroidism, there is a worsening of the lipidemic profile of these patients [40, 51]. In these cases, the coexistence of a bad glycaemic and lipidemic profile could be the onset of acute coronary syndrome and an aggravation of the cardiovascular risk of patients with diabetes mellitus [52].

There is a study performed in Greek adult patients only with type 2 diabetes mellitus examined generally the thyroid dysfunction without precise information about the prevalence of hypothyroidism and/or nodular goiter [6]. This is a clinical study in Greek population of a single center, which compares distinctly patients with type 1 and type 2 diabetes and distinctly the two major thyroid dysfunctions such as hypothyroidism and nodular goiter. It is very interesting that in our study hypothyroidism has a comparable prevalence in patients with type 1 and type 2 diabetes mellitus.

The present study report a high prevalence of hypothyroidism provocated from autoimmunity in patients with T2DM that is comparable with that of T1DM patients in which autoimmunity is expected. The limitations of the study are the small number of T1DM patients [Table 1], but it is known that T1DM patients are always fewer than those with T2DM. Another limitation of the study could be considered that thyroid hormonal tests have not been performed in the same laboratory and the sensitivity of the assays is not known.

In conclusion, screening for thyroid disease among patients with type 2 diabetes mellitus should be routinely considered, as it is found to be an additional commorbidity. Ιf it remains undiagnosed, it could aggravate the clinical course of the disease.

## Figures and Tables

**Table 1:** Characteristics of patients with T1DM and T2DM.

	Type 1 diabetes mellitus	Type 2 diabetes mellitus	*p* ^∗^
1015 patients (60.4% men)	12.6 (%)	87.4 (%)	
Sex (men/women)	58.4/41.6 (%)	52.8/47.2 (%)	
Mean age	47.5 + 1.4 years	67.5 + 0.3 years	**<0.001**
Mean duration of diabetes	23.6 + 1.1 years	15.9 + 0.3 years	**<0.001**
Hypothyroidism	43.5%	37.1%	**ns**
Nodular goiter	18.8%	34.1%	**<0.05**

^∗^The mean difference is significant at the 0.05 level.

**Table 2:** Biochemical and anthropometric measurements in T1DM patients with and without hypothyroidism.

Type 1 diabetes mellitus	Hypothyroidism (±SD)	Without hypothyroidism (±SD)	*p* ^∗^
*N* (128)	56	72	
Age	50.14 ± 15.92	48.6 ± 15.39	NS
Sex (men/women) (%)	33.3/42.2	66.7/57.8	NS
HbA1c	7.74 ± 1.08	7.61 ± 1.33	NS
BMI (Kg/m^2^)	26.77 ± 3.59	26.2 ± 4.59	NS
Chol (mg/dl)	176 ± 32	182 ± 41	NS
HDL (mg/dl)	62 ± 16	53 ± 11	**<0.05**
LDL (mg/dl)	99 ± 28	108 ± 40	NS
TG (mg/dl)	83 ± 33	92 ± 49	NS
Waist (cm)	94 ± 13	93 ± 14	NS
eGFR (mg/ml/min)	99.91 ± 39.52	107.8 ± 41.8	NS
SBP (mmHg)	114.8 ± 11.9	115.5 ± 12.2	NS
Wellness feeling	6.2	6.9	NS

^∗^The mean difference is significant at the 0.05 level.

**Table 3:** Biochemical and anthropometric measurements in T2DM patients with and without hypothyroidism.

Type 2 diabetes mellitus	Hypothyroidism (±SD)	Without hypothyroidism (±SD)	*p* ^∗^
*N* (887)	329	558	
Age	65.44 ± 11.9	67.81 ± 9.19	NS
Sex (men/women) (%)	10.2/43.2	89.8/56.8	<0.001
HbA1c	7.27 ± 1.08	6.98 ± 0.73	<0.01
BMI (Kg/m^2^)	30.28 ± 5.19	29.37 ± 5.06	NS
Chol (mg/dl)	184.97 ± 36.82	168.17 ± 34.86	<0.001
HDL (mg/dl)	51.28 ± 14.17	46.77 ± 11.69	<0.01
LDL (mg/dl)	124 ± 27.54	95.39 ± 28.90	NS
TG (mg/dl)	141 ± 92.35	138 ± 84.46	NS
Waist (cm)	101.64 ± 12.12	102.16 ± 14.04	NS
eGFR (mg/ml/min)	86.68 ± 33.25	84 ± 29.26	NS
SBP (mmHg)	120.67 ± 13.55	123.25 ± 12.25	NS
Wellness feeling	5.3	7.5	<0.001

^∗^The mean difference is significant at the 0.05 level.
